# Effect of *Helicobacter pylori* eradication on metabolic profile: an international, multicenter, case-control study

**DOI:** 10.1186/s12876-022-02604-3

**Published:** 2022-12-08

**Authors:** Pezhman Alavinejad, Eskandar Hajiani, Abazar Parsi, Azam Satari, Mohammad Javad Rezaei, Morteza Nayebi, Ahmad Hormati, Omid Eslami, Mohammed Hussien Ahmed, Quang Trung Tran, Masoud Arshadzadeh, Siamak Baghaei, Samira Mohammadi, Seyed Jalal Hashemi, Alireza Sedaghat

**Affiliations:** 1 Alimentary Tract Research Center, Imam Khomeini Hospital Clinical Research Development Unit, The School of Medicine, Ahvaz Jundishapur University of Medical Sciences, Ahvaz, Iran; 2 Shahid Rajaie Cardiovascular, Medical & Research Center, Iran University of Medical Sciences, Tehran, Iran; 3 Gastrointestinal and Liver Diseases Research Center, Firouzgar Hospital, Iran University of Medical Sciences, Tehran, Iran; 4 Gastroenterology and Hepatology Research Center, Institute of Basic and Clinical Physiology Sciences, Kerman University of Medical Sciences, Kerman, Iran; 5 Lecturer Hepatology Gastroenterology and Infectious Diseases, Faculty of Medicine, Kafrelsheikh University, Cairo, Egypt; 6 Department of Internal Medicine, University of Medicine and Pharmacy, Hue University, Hue, Vietnam; 7 Department of Gastroenterology, Faculty of Medicine, Tehran University of Medical Sciences, Tehran, Iran

**Keywords:** *Helicobacter pylori*, Metabolic profile, Eradication

## Abstract

**Background:**

As a gram-negative and microaerophilic bacterium, *Helicobacter pylori* (HP) is the main cause of chronic gastritis. Therefore, considering the high prevalence of HP infection worldwide, as well as the increasing prevalence of metabolic disorders, the present study aimed to investigate the relationship between HP infection eradication and metabolic profile.

**Methods:**

This prospective case-control study was performed on patients with HP infection whom referred to 7 medical centers in 3 countries (Iran, Egypt, and Vietnam) in 2020–2021. The metabolic profile of all of the participants evaluated before starting of treatment for HP eradication and 3 months after the treatment. Then changes of metabolic profile compared between those with successful HP eradication (group A) and subjects who failed to eradicate (group B).

**Results:**

Overall, 199 patients, including 93 male (46.7%) with the mean age of 44.5 years (18–93 years) included. Based on response to treatment, the participants allocate into group A (those who respond to HP eradication): 164 cases (82.42%); or group B as those who failed to achieve eradication (35 cases, 17.58%). Racially 86.9% of participants were Caucasian and 89% diagnosed as non-ulcer dyspepsia (NUD). The most prevalent comorbidity include hypertension (11.5%) and hyperlipidemia (10%) which were more prevalent in group B (*P* = 0.002). Three months after therapy, average weight of participants among those who achieved eradication (group A) decreased from 73.1 to 71.4 kg (*P* = 0.01), but in comparison with group B, was non-significant (*P* = 0.171). The BMI of patients before and after treatment did not show any significant differences. The biochemical parameters of patients before and after treatment were not significantly different regardless of treatment success (*P* > 0.05). The levels of total cholesterol and VLDL cholesterol after treatment were not significantly different from baseline values in two groups. HDL and LDL cholesterol levels before and after treatment in the resistant group were significantly higher than the responding group. Average serum TG level decreased significantly after treatment in the group A (*P* < 0.0001), in contrast to the resistant group (*P* = 0.356). The liver transaminases (AST and ALT) before and after treatment were not significantly different between the two groups (*P* > 0.05). The results of logistic regression showed that the eradication of infection has no significant affect any of the metabolic profile parameters.

**Conclusion:**

HP infection treatment in individuals without significant metabolic disorders does not affect metabolic parameters up to 3 months after eradication. HP eradication among subjects with several comorbidities mandates eradication protocol intensification to avoid treatment failure.

## Introduction

*Helicobacter pylori* (HP) infection is one of the most common infections globally and involves more than 50% of the human population. Moreover, it has a higher prevalence in developing countries [1–3]. Epidemiologic studies have reported prevalence of this infection about 24%, 37%, 47%, 55%, 63%, and 79% among the populations of Oceania, North America, Europe, Asia, Latin America, and Africa respectively [4, 5].

HP infection could be associated with several upper gastrointestinal problems, such as chronic gastritis, peptic ulcer and gastric cancer or lymphoma. Moreover, recent studies have reported a potential association between HP infection and some non-gastric problems, such as metabolic syndrome risk factors, atherosclerosis, and cardiovascular diseases, including myocardial infarction and stroke [6–10]. HP induced gastrointestinal inflammation may impair the uptake of glucose and lipids, resulting in altered lipid and glucose metabolisms. Moreover, some studies have reported a significant relationship between HP infection and increased risk of metabolic disorders. Such relations also state by systemic inflammation and altered leptin and ghrelin levels caused by HP infection [11–15].

According to recent evidences, abnormal lipid profile, including Low-Density Lipoprotein (LDL) and High-density Lipoprotein (HDL), are important metabolic risk factors for cardiovascular diseases and HP infection is associated with altered serum lipid levels [16]. Some studies have even reported significant relationships between HP infection and altered HDL, LDL, total cholesterol and Triglyceride (TG) levels [17]. One possibility is HP mediated atherogenic property by its effects on lipid and glucose metabolisms. Some evidences reported that successful eradication of HP infection can improve gastric mucosal damage and prevent subsequent gastric cancer and other related complications. However, the effect of HP eradication on lipid profile, glucose levels, and other parameters of the metabolic profile has not illustrated yet [18, 19].

A recent meta-analysis showed that HDL and TG levels improved significantly following HP eradication compared to baseline values before the treatment. However, LDL levels did not affected significantly by treatment [20]. On the other hand, an older meta-analysis reported that the HP eradication has no significant effect on serum lipid profile and insulin resistance [21, 22]. Therefore, considering the high prevalence of HP infection in worldwide, as well as the increasing prevalence of metabolic disorders, the present study aimed to investigate the relationship between HP infection eradication and metabolic profile.

## Methods

This prospective case-control study conducted on patients with HP infection who attend in outpatient clinics of seven referral medical centers in three countries (Iran, Egypt, and Vietnam) in 2020–2021. The study approved by the ethics committee of Ahvaz Jundishapur University of Medical sciences (IR.AJUMS.HGOLESTAN.REC.1400.089) based on the principles of the Declaration of Helsinki for human research. Firstly, the purpose and method of conducting the study explained to the participants. Then, eligible patients requested to sign a written consent if they wished, before entering the study. The pertained ethic number and IRB approved and accepted by all of the participating centers.

Inclusion criteria include age higher than 18 years, clinical diagnosis as peptic ulcer disease (PUD), non-ulcer dyspepsia (NUD), or intestinal metaplasia, confirmation of HP infection in biopsy sample by pathological report and positive fecal antigen test, and signing the consent form to participate in the study. Exclusion criteria include presence of serious underlying diseases including active malignancies, sever heart failure, uncontrolled diabetes mellitus, sever renal failure, advanced chronic liver disease, viral hepatitis, history of any gastric surgery, current pregnancy or lactation, and previous history of HP eradication during last 6 months before study.

At the beginning, the demographic characteristics of patients (age, sex, medical history, body mass index (BMI), smoking) recorded through a checklist. The metabolic profile of participants including Fasting Blood Sugar (FBS), alanine aminotransferase (ALT), aspartate aminotransferase (AST), alkaline phosphatase (ALP), HDL-C, LDL-C, cholesterol, TG, BUN and Cr measured and determined. Then all of the patients treated for HP infection with one of the existing protocols based on responsible physician choice. The participants followed by weekly phone calls and requested to report any side effects or complication related to medical therapy.

All of the participants received eradication regimen for 2 weeks, then the therapy with PPI continued for four more weeks (totally 6 weeks). Two weeks after the end of the treatment period and discontinuation of the drugs (week 8), a stool antigen test (SATs) performed to prove the eradication and the effectiveness of the treatment. Based on success of eradication, the participant allocated to group A (those who achieved eradication) and group B (those who failed eradication). Three months after the end of treatment, the Metabolic profile of participants including blood glucose, lipid profile, and liver transaminases measured again and any changes compared between two groups.

### Statistical methods

Statistical analysis performed by SPSS software (SPSS Inc., Chicago, IL, USA) version 22. For quantitative variables, the mean and median determined, and the data scatter from the standard deviation and Interquartile range (IQR) used. In qualitative variables, frequency and percentage used to describe the data. The normality of the data checked by Kolmogorov-Smirnov test and the Q-Q diagram. Non-parametric Mann-Whitney U and Chi-squared tests applied to analyze the results, and Wilcoxon test used to compare the changes of different parameters before and after treatment. Logistic regression (LR) used and the level of significance in the tests was considered 0.05.

## Results

**Fig. 1:**
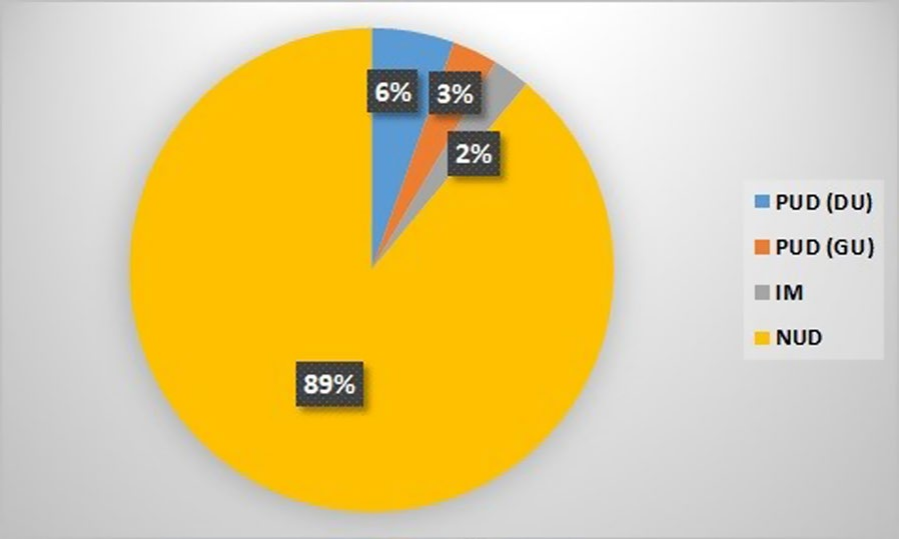
Clinical diagnosis of participants (*PUD* Peptic ulcer disease; *DU* Duodenal ulcer; *GU* Gastric ulcer; *IM* Intestinal metaplasia; *NUD* Non-ulcer dyspepsia)

This study performed on 199 patients, including 93 male (46.7%) with the mean age of 44.57 ± 13.92 years (18–93 years) (Table 1). Based on response to treatment, the participants allocate into group A (those who respond to HP eradication): 164 cases (82.42%); or group B as those who failed to achieve eradication (35 cases, 17.58%). Racially 86.9% of participants were Caucasian. 89% of participants diagnosed as non-ulcer dyspepsia (NUD) (Fig. 1). The most prevalent comorbidity include hypertension (11.5%) and hyperlipidemia (10%) (Table 2) and prevalence of them was higher in group B who failed eradication (*P* = 0.002) (Table 1). There was no significant difference in the frequency of HP induced GI disease between two groups (*P* = 0.06) (Table 1).

**Table 1:** Baseline characteristics of patients in the two groups

Variables	Group A (N = 164)	Group B (N = 35)	*P*-value
Age (range)	42 (34–52)	43.5 (35.75–55.5)	0.291*
Gender, Male N (%)	75 (49.4)	12 (35.3)	0.165**
Race, N (%)			
Caucasian	144 (87.8)	29 (82.8)	0.182**
Arab	14 (8.5)	4 (11.8)	
Turkish	4 (2.6)	1 (2.9)	
Asian	2 (1.3)	0	
African	0	1 (2.9)	
Smoke, N (%)	35 (22.6)	5 (14.7)	0.309**
Comorbidity, N (%)	32 (19.5%)	15 (42.8%)	0.002**
Cause of eradication, N (%)			
NUD	150 (91.4%)	27 (77.1%)	0.06**
DU	6 (3.6%)	5 (14.2%)	
GU	4 (2.4%)	2 (5.7%)	
IM	4 (2.4%)	1 (2.8%)	

*DU* Duodenal ulcer; *GU* Gastric ulcer; *IM* Intestinal metaplasia; *NUD* Non-ulcer dyspepsia

*Mann-Whitney U, **Chi-squared

**Table 2:** Concomitant disorders of participants beside HP infection; some of the participants involved with more than one disorder

Concomitant disorders	Number	Percent
HTN	23	11.50
HLP	20	10
IHD	16	8
DM	9	4.50
CLV	3	1.50
CRF	2	1
Depression	1	0.50
Hypothyroidism	1	0.50

*HTN* Hypertension; *HLP* Hyperlipidemia; *IHD* Ischemic heart disease; *DM* Dmellitus; *CLV* Chronic liver disease; *CRF* Chronic renal failure

The most common therapeutic protocols include amoxicillin-based dual therapy and clarithromycin base triple or quadruple regimens (Table 3). The success rate of eradication among those without comorbidity was 86.8% versus 68% in subjects with any comorbidity (*P* = 0.0031).

**Table 3:** HP therapeutic regimens

Therapeutic regimen	Group A, n (%)	Group B, n (%)	*P* value
High dose Amoxicillin (dual therapy)	75 (45.73)	14 (40)	0.296
Clarithromycin base (triple or quadruple)	47 (28.6)	7 (20)	0.536
Levofloxacin base	3 (1.8)	4 (11.7)	0.005
BOMT	3 (1.8)	0	0.420
BOMA	3 (1.8)	8 (22.8)	0.0001
Miscellaneous	32 (19.5)	2 (5.7)	0.042

*A* Amoxicillin; *B* Bismuth subcitrate; *M* Metronidazole; *O* Omeprazole; *T* Tetracycline

Three months after therapy, average weight of participants among those who achieved eradication (group A) decreased from 73.1 to 71.4 kg (*P* = 0.01), but in comparison with group B, this weigh reduction was non-significant (*P* = 0.5) (Table 4). The BMI of patients before and after treatment did not show any significant difference**s.** The biochemical parameters of patients before and after treatment were not significantly different regardless of treatment success (*P* > 0.05) (Table 4). The levels of total cholesterol and VLDL cholesterol after treatment were not significantly different from baseline values in two groups. HDL and LDL cholesterol levels before and after treatment in the resistant group were significantly higher than the responding group. Average serum TG level decreased significantly after treatment in the group A (*P* < 0.0001), but in the resistant group, no significant change was observed in TG serum level (*P* = 0.356) (Table 4).

**Table 4:** Changes in anthropometric, biochemical, lipid profiles, and liver function parameters before and three months after treatment to eradicate *H. pylori* infection based on response to treatment

Variables, mean ± S.D (Min-Max)	Response to treatment (N = 164)	Resistant to treatment (N = 35)	*P*-value*
Anthropometric parameters			
Weight, Kg			
Before treatment	73.18 ± 14.67 (48–135)	73.74 ± 15.53 (45–110)	0.685
After treatment	71.46 ± 12.86 (33–125)	73.44 ± 15.10 (45–110)	0.500
	0.011**	0.568**	
BMI, Kg/m ^2^			
Before treatment	25.62 ± 4.33 (18.2–43)	27.13 ± 6.32 (16.5–42.97)	0.188
After treatment	25.54 ± 4.41 (18.2–45)	26.9 ± 6.16 (16.5–42.97)	0.236
	0.074**	0.172**	
Biochemical parameters			
Hb, g/dl			
Before treatment	12.73 ± 2.05 (8.5–17.5)	13.10 ± 1.96 (8.9–16.0)	0.336
After treatment	13.4 ± 3.39 (8.5–17.5)	12.97 ± 2.03 (8.6–17)	0.846
	0.086**	0.083**	
PLT, µL 10^3^			
Before treatment	295.37 ± 84.21 (121–584)	284.48 ± 82.47 (150–468)	0.507
After treatment	292.05 ± 81.75(120–484)	283.90 ± 73.85 (150–391)	0.607
	0.937**	0.016**	
MCV, fL			
Before treatment	83.61 ± 5.00 (66–98)	83.86 ± 5.12 (74–98)	0.989
After treatment	83.96 ± 5.51 (58–98)	83.53 ± 4.79 (71–95)	0.934
	0.935**	0.278**	
FBS, mg/dl			
Before treatment	96.17 ± 23.12 (63–302)	97.14 ± 15.53 (68–141)	0.455
After treatment	94.22 ± 15.12 (61–170)	97.60 ± 14.13 (71–140)	0.395
	0.759**	0.346**	
BUN, mmol/L			
Before treatment	15.34 ± 6.06 (2.0–38.0)	17.04 ± 5.94 (7.0–30)	0.094
After treatment	16.59 ± 8.70 (1.6–84)	17.91 ± 6.47 (7.0–30)	0.427
	0.073**	0.782**	
Cr, mg/dL			
Before treatment	1.77 ± 1.78 (0.2–8.0)	1.56 ± 1.30 (0.6–6.0)	0.733
After treatment	1.76 ± 1.73 (0.2–8.0)	1.51 ± 1.35 (0.6–6.5)	0.873
	0.522**	0.428**	
Calcium, mg/dL			
Before treatment	8.61 ± 0.83 (5.7–10.5)	9.01 ± 0.64 (7.20–9.80)	0.061
After treatment	8.79 ± (7.5–10.5)	9.15 ± 0.57 (8.10–10.10)	0.038
	0.549**	0.634**	
TSH, mIU/L			
Before treatment	2.62 ± 1.34 (0.4–8.5)	2.29 ± 1.12 (0.3–5.50)	0.591
After treatment	2.47 ± 1.09 (0.6–6.1)	2.25 ± 1.25 (0.3–5.75)	0.496
	0.299**	0.549**	
25-OH-VitD, ng/mL			
Before treatment	26.80 ± 27.34 (6.0–65.0)	28.56 ± 13.48 (13–60)	0.768
After treatment	29.02 ± 9.84 (3.7–56.0)	30.93 ± 12.76 (16–56)	0.894
	0.210**	0.057**	
Lipid profiles			
TC, mg/dL			
Before treatment	163.85 ± 37.68 (77–275)	175.14 ± 38.37 (91–265)	0.137
After treatment	166.37 ± 35.75 (80–289)	173.51 ± 38.83 (115–279)	0.322
	0.837**	0.754**	
TG, mg/dL			
Before treatment	155.65 ± 56.20 (56–253)	152.09 ± 51.06 (96.5–253.5)	0.313
After treatment	150.94 ± 48.90 (39–271)	150.26 ± 43.41 (85–280)	0.472
	< 0.0001**	0.468**	
HDL-C, mg/dL			
Before treatment	39.58 ± 17.98 (18–131)	44.41 ± 12.75 (23–80)	0.013
After treatment	40.85 ± 11.57 (18–97)	45.35 ± 11.89 (27–74)	0.043
	0.121**	0.624**	
LDL-C, mg/dL			
Before treatment	87.67 ± 29.81 (25–195)	103.65 ± 35.51 (20–165)	0.016
After treatment	86.82 ± 28.71 (25–167)	103.85 ± 35.75 (24–185)	0.027
	0.457**	0.449**	
VLDL-C, mg/dL			
Before treatment	43.34 ± 12.94 (11 − 85)	42.46 ± 17.71 (11–95)	0.327
After treatment	39.52 ± 10.27 (7–61)	40.71 ± 15.9 (15–68)	0.549
	0.265**	0.116**	
Liver function parameters			
AST, U/L			
Before treatment	27.02 ± 10.09 (12–68)	26.80 ± 16.36 (9–90)	0.174
After treatment	28.10 ± 12.62 (12–99)	26.46 ± 10.93 (10–71)	0.341
	0.194**	0.059**	
ALT, U/L			
Before treatment	30.59 ± 12.65 (10–69)	30.0 ± 16.96 (10–79)	0.378
After treatment	31.21 ± 12.74 (10–88)	31.23 ± 13.36 (11–74)	0.945
	0.502**	0.096**	
ALP, IU/L			
Before treatment	210.39 ± 80.92 (61–420)	207.51 ± 68.63 (61–357)	0.880
After treatment	214.10 ± 72.42 (61–488)	195.86 ± 64.51 (61–401)	0.554
	0.804**	0.026**	
Total bilirubin, mg/dL			
Before treatment	0.97 ± 1.28 (0.1–14.0)	0.81 ± 0.52 (0.2–2.50)	0.898
After treatment	0.94 ± 1.39 (0.1–6.0)	0.83 ± 0.51 (0.2–2.5)	0.460
	0.051**	0.566**	
Direct bilirubin, mg/dL			
Before treatment	0.95 ± 1.39 (0.1–14.0)		0.436
After treatment	0.37 ± 0.69 (0.1–6.0)	0.25 ± 0.23 (0.1–1.10) 0.24 ± 0.22 (0.1–1.10)	0.648
	0.891**	0.979**	

*BMI* Body mass index; *Hb* Hemoglobin; *PLT* Platelet count; *MSV* Mean corpuscular volume; *FBS* Fasting blood sugar; *BUN* Blood urea nitrogen; *Cr* Creatinine; *TSH* Thyroid-stimulating hormone; *25-OH-VitD* 25-hydroxyvitamin D (25(OH)D); *TC* Total cholesterol; *TG* Triglyceride, *HDL-C* High-density lipoprotein cholesterol; *LDL-C* Low density lipoprotein cholesterol; *VLDL-C* Very low density lipoprotein cholesterol; *AST* Aspartate aminotransferase; *ALT* Alanine aminotransferase; *ALP* Alkaline phosphatase

*Mann-Whitney U, **P-value, Wilcoxon test

The liver transaminases (AST and ALT) before and after treatment were not significantly different between the two groups (*P* > 0.05) (Table 4). The results of logistic regression showed that the eradication of infection has no significant affect any of the metabolic profile parameters. The adjusted logistic regression model based on various variables (including age, sex, race, nationality, underlying disease, type of HP induced GI disease, therapeutic regimen, and smoking), showed that eradication of HP infection had no significant effect on the any of the patients’ metabolic profile parameters (Table 5).

**Table 5:** Logistic regression to predict the effect of HP eradication on metabolic profile

Variables	Non-adjusted analysis		Adjusted analysis	
	OR (95% CI)	* P*-value	OR (95% CI)	* P*-value
Weight	0.96 (0.9–1.3)	0.333	0.878 (0.9–1.02)	0.171
BMI	1.05 (0.82–1.35)	0.695	1.01 (0.83–1.24)	0.876
FBS	0.99 (0.96–1.01)	0.485	0.98 (0.96–1.01)	0.42
BUN	1.07 (0.93–1.23)	0.301	1.15 (0.96–1.38)	0.11
Cr	0.76 (0.16–3.43)	0.723	0.56 (0.08–3.71)	0.553
25-OH-VitD	0.98 (0.92–1.04)	0.609	1 (0.93–1.08)	0.879
TC	1 (0.99–1.01)	0.58	1 (0.99–1.01)	0.644
TG	1 (0.99–1.01)	0.783	1 (0.99–1)	0.929
HDL-C	1 (0.99–1.01)	0.287	1 (0.99–1.01)	0.526
LDL-C	0.99 (0.98–1.01)	0.763	0.99 (0.98–1.01)	0.642
VLDL-C	0.96 (0.87–1.05)	0.386	0.95 (0.85–1.06)	0.366
AST	1.02 (0.97–1.06)	0.357	1 (0.96–1.05)	0.742
ALT	0.99 (0.94–1.03)	0.678	0.98 (0.94–1.03)	0.568
ALP	1 (0.99–1)	0.332	1 (0.99–1)	0.517

*OR adj* Adjusted odds ratio; *CI* Confidence interval; *BMI* Body mass index; *Hb* Hemoglobin; *PLT* Platelet count; *MSV* Mean corpuscular volume; *FBS* Fasting blood sugar; *BUN* Blood urea nitrogen; *Cr* Creatinine; *TSH* Thyroid-stimulating hormone; *25-OH-VitD* 25-hydroxyvitamin D (25(OH)D); *TC* Total cholesterol; *TG* Triglyceride; *HDL-C* High-density lipoprotein cholesterol; *LDL-C* Low density lipoprotein cholesterol; *VLDL-C* Very low density lipoprotein cholesterol; *AST* Aspartate aminotransferase; *ALT* Alanine aminotransferase; *ALP* Alkaline phosphatase

*adjusting for age, sex, race, comorbidities (DM, HTN, smoking); diagnosis and treatment regimen

## Discussions

According to findings of the current study, successful HP eradication could be accompany by a significant decrease in serum TG levels, 3 months after the treatment compared to baseline values while in Logistic regression model, it was non-significant (Table 5, *P* = 0.929). However, there was no difference between the responders and refractory cases in other metabolic profile parameters. Moreover, the levels of other metabolic profile parameters would not significantly change up to 3 months after the treatment. A study by Adachi et al. in 2018 reported the improving effect of HP infection eradication on the lipid metabolism of the affected patients. They compared the lipid profiles of the patients with and without HP infection and showed significantly lower HDL levels in the patients with HP [23]. Likewise, patients with successful HP eradication had higher HDL levels and lower total cholesterol, LDL, and TG levels compared to those with persistent HP infection.

A meta-analysis showed increased HDL and TG levels in the patients with HP infection following eradication compared to pre-treatment values. However, serum LDL levels were not significantly changed [20]. These findings were compatible with the results of current study, reporting significantly reduced TG levels following infection eradication. Moreover, a cohort study reported that the patients with successful HP infection eradication had significantly decreased HDL and increased LDL levels 1.5 years after the treatment compared to the group who were positive for HP infection [24]. A potential explain could be better appetite due to gastritis management after HP eradication.

According to another study, eradication of HP infection could significantly decrease the TG levels while causing a slight increase in HDL levels following 1 year and 8 weeks from commencing the treatment. However, other metabolic parameters were not significantly changed. On the other hand, a study by Maruyama et al. reported no significant changes in total cholesterol, TG, LDL, and HDL levels after HP infection eradication compared to values before the treatment, which could explain by the use of statins and anti-HP regimen in the patients [25]. Another study by Nam et al. reported the improving effect of HP treatment on the lipid profile of the affected patients, including increased HDL and decreased LDL levels, after a 2 year follow-up period [26].

A study by Haeri et al. (2018) in Iran did not show a significant difference in LDL and HDL levels between the patients with and without HP infection [19]. However, Ansari et al. (2010) reported significantly lower HDL and Apolipoprotein A1 (Apo A1) levels and higher total cholesterol, TG, LDL, Apolipoprotein B (Apo B), Alkaline Phosphatase (ALP), cholesterol-to-HDL ratio, and LDL-to-HDL ratio in the patients with HP infection compared to the control group [27]. Considering all these findings, the effect of HP infection on metabolic profile, including the lipid profile, is still controversial. Therefore, further studies are mandatory to confirm the changes in lipid profile due to HP infection, as well as the improving effect of HP eradication on lipid profile. On the other hand, the relation between HP infection and cardiovascular events and stroke have reported in several investigations [9, 10] and could be explained by potential involvement of HP infection in atherosclerosis pathogenesis by inducing local or systemic inflammation and subsequently stimulating plaque progression and instability [8, 11–13].

There was no significant intergroup or intragroup difference in other biochemical parameters, including hemoglobin, platelet, Mean Corpuscular Volume (MCV), creatinine, calcium, Thyroid-Stimulating Hormone (TSH), and vitamin D levels. However, a recent study by Shafrir et al. (2021) reported a relationship between vitamin D levels and HP infection, showing significantly lower vitamin D levels in patients with HP infection compared to normal non-infected individuals [28]. Moreover, the group who responded to treatment had significantly higher vitamin D levels compared to the unresponsive group. Therefore, it is possible that HP infection be able to disturb vitamin D absorption by induction of duodenal inflammation although this issue needs further verification in future studies.

The present study showed a non-significant decreased BMI in the responsive group 3 months after the treatment compared to the pre-treatment values (*P* = 0.074). Therefore, there was no concern about obesity and weight gain in the present study. Moreover, a study by Liou et al. (2019) showed that HP infection eradication could significantly reduce the BMI of the patients after 8 weeks of eradication treatment [29]. However, a study by Zojaji et al. (2013) did not show the significant effect of HP infection on BMI [30]. These findings were compatible with the present study and same as some other studies with similar results [31, 32]. Although, they were incompatible with several studies showing a significant BMI increase after HP infection eradication. Therefore, there is a potential negative relationship between HP infection and obesity [25, 33–35], and the controversy between different studies can be due to different populations and methods.

According to our results, there was no significant intergroup or intragroup difference in the Fasting Blood Sugar (FBS) of the patients. Therefore, eradication of HP infection had no significant effect on the patients’ FBS. However, some studies have shown the improving effect of HP eradication on FBS. For example, a study by Dogan et al. (2015) on 370 patients with normal glucose levels showed the improving effect of successful eradication of HP on FBS, HbA1c, and insulin resistance (IR-HOMA) of the patients 6 months after the treatment compared to pre-treatment values [22]. They concluded that HP eradication could significantly improve the FBS levels and insulin resistance in patients with normal glucose levels which is in contrast to finding of current study.

An interesting finding in current work is higher prevalence of comorbidities such as hypertension and hyperlipidemia among resistant cases who fail to achieve HP eradication (*P* = 0.02). This issue means higher probability of successful HP eradication among those without comorbidity (86.8% vs. 68%, *P* = 0.0031). If this issue prove in future studies, can interprets as necessity of HP eradication regimens intensification by prolongation of the course or using higher doses of antibiotics among those with several comorbidities.

The limitation of current work was short period of patients follow up (3 months) and it is postulated that longer follow up time be able to help further elucidation of any relation between HP infection and metabolic profile.

## Conclusion

HP infection eradication significantly reduced the serum TG levels in the patients with successful eradication. However, it had no significant effect on other metabolic profile parameters up to 3 months after eradication. On the other hand, HP eradication among subjects with several comorbidities mandates eradication protocol intensification to avoid treatment failure.

## Data Availability

The DATA would be available based on personal request to corresponding author.
